# Genomic and clinicopathological features of lung adenocarcinomas with micropapillary component

**DOI:** 10.3389/fonc.2022.989349

**Published:** 2022-11-15

**Authors:** Peng Li, Lu Liu, Dong Wang, Ronghua Yang, Yunpeng Xuan, Yudong Han, Jinglong Wang, Lijie Guo, Liwen Zhang, Shanshan Zhang, Yongjie Wang

**Affiliations:** ^1^ Department of Thoracic Surgery, The Affiliated Hospital of Qingdao University, Qingdao, Shandong, China; ^2^ Department of Anesthesiology, The Affiliated Hospital of Qingdao University, Qingdao, Shandong, China; ^3^ Medical Department, OrigiMed Co., Ltd, Shanghai, China

**Keywords:** lung adenocarcinoma, micropapillary component, next-generation sequencing, clinicopathological features, prognosis

## Abstract

**Background:**

Lung adenocarcinoma (LA) with a micropapillary component (LAMPC) is a histological subtype of lung cancer that has received increasing attention due to its correlation with poor prognosis, and its tendency to recur and metastasize. At present, comprehensive genomic profiles and clinicopathological features for LAMPC remain unclear and require further investigation.

**Methods:**

From September 2009 to October 2020, a total of 465 LAMPC patients were recruited and divided into four groups according to MPC proportions, and the correlations between varying proportions of MPCs and clinicopathological characteristics were analyzed. Twenty-nine (29) LAMPC patients and 89 LA patients without MPC (non-MPC) that had undergone NGS testing were selected for further study The comprehensively analyze genomic variations and the difference between LAMPC and MPC were determined. In addition, Gene alterations of LAMPC between Chinese and Western populations were also compared using cBioPortal data.

**Results:**

A higher proportion of MPCs, associated with higher tumor stage, pleural invasion, and vascular tumor thrombus formation, was determined in LA patients. Compared to non-MPC patients, LAMPC patients were determined to have a lower frequency of single nucleotide variants and a higher frequency of insertion-deletion mutations. Mutations in *TP53*, *CTNNB1*, and *SMAD4*, and *ALK* rearrangements/fusions were significantly more frequent in LAMPC patients. *ERBB2* mutations were only detected in non-MPC patients. Gene mutations in the Wnt pathway were significantly more common in LAMPC patients as compared to non-MPC patients. *ALK* fusions were more prevalent in younger patients. Patients with *KRAS* or *LBP1B* mutations had significantly larger tumor diameters than patients with wild-type *KRAS* or *LBP1B*. Patients with *KRAS* mutations were more likely to develop vascular tumor thrombus. Using the cBioPortal public database, we determined that mutations in *EGFR* were significantly higher in Chinese patients than in a Memorial Sloan Kettering Cancer Center (MSKCC) Western cohort. *ALK* fusions were exclusively detected in the Chinese cohort, while mutations in *KEAP1* and *NOTCH4* were only detected in the MSKCC cohort. Our analysis of signaling pathways revealed that Wnt pathway gene mutations were significantly higher in the Chinese cohort.

**Conclusion:**

LA patients with higher proportions of MPCs were determined to have a higher tumor stage, pleural invasion, and vascular tumor thrombosis formation. We comprehensively analyzed the genomic mutation characteristics of LAMPC patients and identified multiple, novel MPC-related gene alterations and pathway changes. Our data provide further understanding of the nature of the LAMPC and potential drug-targeted gene alterations, which may lead to new therapeutic strategies.

## Introduction

Lung cancer is the leading cause of cancer death in China, and lung adenocarcinoma (LA) is the most prevalent histological type of cancer amongst non-small cell lung cancers, accounting for nearly 50% of worldwide cases (1–3). In 2011, the International Association for the Study of Lung Cancer (IASLC), the American Thoracic Society (ATS), and the European Respiratory Society (ERS) proposed a new system, in which LA is classified into the following five subtypes according to main histological patterns: lepidic, acinar, papillary, micropapillary, and solid. In 2015, the same classification system was adopted by the World Health Organization (4–6). LA with a micropapillary component (LAMPC), which occurs when a micropapillary pattern accounts for > 1% of an entire tumor, is an established histological subtype of lung cancer. LAMPC is characterized by the presence of tumor cells with floating ring-shaped glandular structures within the alveolar space. Due to its high malignancy and aggressiveness, as well as its poor prognosis and tendency to recur and metastasize, LAMPC has received increasing attention (7–9). However, differences in clinicopathological features and proportions of MPC in LA patients remain unclear.

A multivariate analysis revealed that MPC is an independent risk factor for lower recurrence-free survival and overall survival (OS) (10–12). Currently, surgical resection, chemotherapy, and targeted therapy are the main treatment options for pulmonary micropapillary adenocarcinoma. Patients with LAMPC have increased local recurrence when treated with limited resection (13) and micropapillary predominant LA in stage is benefits from adjuvant chemotherapy (14). Micropapillary-predominant adenocarcinomas respond well to platinum-based chemotherapy and EGFR tyrosine kinase inhibitors (TKI) (15, 16). While micropapillary-predominant adenocarcinomas benefit, to a greater extent, from adjuvant chemotherapy than other histological subtypes, they do not benefit from adjuvant radiotherapy (17). Campos-Parra et al. (18) determined that micropapillary-predominant lung adenocarcinomas respond better to chemotherapy, resulting in a better OS. Another study revealed that LAMPC patients with *EGFR* mutations treated with EGFR-TKIs had a significantly better post-recurrent survival than patients who did not receive TKI treatment (19).

In this study, we first analyzed correlations between different proportions of MPC and clinicopathological features in LAMPC patients, and then used next generation sequencing (NGS) to comprehensively analyze the genomic characteristics of LAMPC patients. We identified multiple new MPC-related gene alterations and pathway changes which may help us better understand the nature of this cancer subtype and determine potential drug-targeted gene alterations, potentially leading to new therapeutic strategies.

## Materials and methods

### Patients

From September 2009 to October 2020, a total of 465 LAMPC patients were recruited from the Department of Thoracic Surgery at the Affiliated Hospital of Qingdao University. The patient cohort included 215 males and 250 females with a median age of 60 years old. Most patients had an early stage tumor and approximately 70% of patients were in Stages I-II. Patients were divided into four groups based on the proportions of MPC. We collected data in order to analyze the correlation between different proportions of MPC and clinicopathological characteristics. To comprehensively analyze genomic variations, 31 LAMPC samples (from 29 LAMPC patients) and additional 89 non-MPC that had undergone NGS testing were selected. Our study was approved by the Ethics Committee of the Affiliated Hospital of Qingdao University (Ethics No.: QYFYKYLL: 999811920), and all patients signed an informed consent form.

### Clinicopathological data

Clinicopathological data was collected from 465 patients with LAMPC and included patient gender, age at diagnosis, tumor size, histological subtype, morphological characteristics, clinical stage, pleural invasion, and vascular tumor thrombus invasion and spread through air spaces (STAS). Histological subtypes of LA were defined according to standard criteria provided by the 2011 International Association for the Study of Lung Cancer (IASLC), the American Thoracic Society (ATS), and the European Respiratory Society (ERS). TNM staging was based on the 8^th^ Edition of the IASLC.

### Sample collection, DNA extraction, and genomic mutation detection

Formalin-fixed, paraffin-embedded (FFPE) tumor tissue and matched blood samples were collected, and genomic DNA was prepared using a commercial kit (Qiagen, Hilden, Germany). DNA concentrations were measured using Qubit. Patient samples were sequenced using the Illumina (Illumina Inc., CA, USA) NGS platform, with an average sequencing depth of > 50x. Based on the sequencing of at least 420 genes (420−638), a total of 296 genes within the intersection portion of each panel were selected for analysis. Genomic alterations, including single nucleotide variants (SNVs), short and long insertion-deletion variations (indels), copy number variations (CNVs), and gene rearrangements, were included in the analysis.

### Statistical analyses

Statistical analyses were conducted using the R Statistical Software package (R Foundation for Statistical Computing, Vienna, Austria). Data for categorical variables are presented as frequencies and percentages, while data for continuous variables are reported as medians and percentiles. A student *t*-test or a Wilcoxon rank test were used to compare two continuous data sets. Chi-square tests or Fisher’s exact tests were used to compare two sets of categorical data. A 2-sided *p* value less than 0.05 was considered statistically significant.

## Results

### The correlation analysis between MPC proportions and clinicopathological features

**Figure 1:**
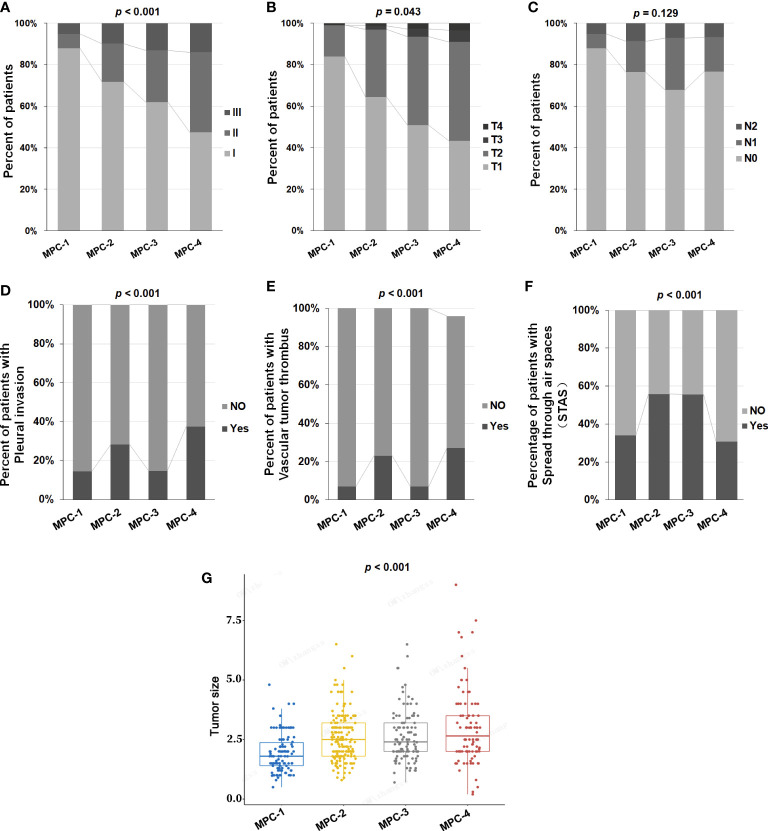
The correlation between different proportions of MPC and clinicopathological features in LAMPC patients. The correlation between different proportions of MPC and **(A)** stage, **(B)** T status, **(C)** N status, **(D)** pleural invasion, **(E)** vascular tumor thrombus, **(F)** spread through air spaces (STAS) and **(G)** tumor size. p< 0.05 was considered statistically significant, and p < 0.01 was considered more statistically significant.

A total of 465 LAMPC patients were divided into four groups according to proportions of MPC. One-hundred and six (106) patients had an MPC below 5% (MPC-1), 163 patients had an MPC of 5-20% (MPC-2), 108 patients had an MPC of 20-50% (MPC-3), and 88 patients had an MPC of at least 50% (MPC-4). We explored the correlation between different proportions of MPC and clinicopathological features, and determined that the proportions of MPC correlated with tumor stage. A higher proportion of MPC correlated with a more advanced stage (*p* < 0.05) (**Table 1** and **Figures 1A, B**), but did not correlate with N status (*p* = 0.129) (**Table 1** and **Figure 1C**). The proportion of MPC was significantly correlated with pleural invasion and vascular tumor thrombus formation. In other words, patients with a higher proportion of MPC are more likely to have pleural invasion and vascular tumor thrombus (*p* < 0.001) (**Table 1** and **Figures 1D, E**). We also assess the correlation between STAS status and MPC, and observed that STAS was found in 214 of 465 (46.0%) cases, and STAS was found to be significant more prevalent in MPC-2 and MPC-3 groups compared to MPC-1 and MPC-4 groups (*p <*0.001, **Table 1** and **Figure 1F**). Larger tumor diameters were also associated with a higher proportion of MPC (*p* < 0.001) (**Table 1** and **Figure 1G**). No significant correlation was determined between MPC and gender or age (*p* > 0.05).

**Table 1:** Correlation analysis between different micropapillary content and clinicopathologic features.

			Total (n=465)	MPC-1 (n=106)		MPC-2 (n=163)	MPC-3 (n=108)		MPC-4 (n=88)		p-value
**Age**											0.31
Median age, years (range)		60 (26-81)		58 (39-80)		60 (26-81)	60 (35-77)			62 (30-78)	
**Gender**											0.063
Male		215 (46.2%)		54 (50.9%)		62 (38.0%)	57 (52.8%)			42 (47.7%)	
Female		250 (53.8%)		52 (49.1%)		101 (62.0%)	51 (47.2%)			46 (52.3%)	
**Tumor Size**											<0.001
Median (range)		2.3 (0.2-9)		1.8 (0.5-4.8)		2.5 (0.8-6.5)	2.4 (0.7-6.5)			2.7 (0.2-9)	
**Stage**											<0.001
I		247 (53.3%)		51 (48.1%)		117 (71.8%)	52 (48.1%)			27 (30.7%)	
II		77 (16.6%)		4 (3.8%)		30 (18.4%)	21 (19.4%)			22 (25.0%)	
III		38 (8.2%)		3 (2.8%)		16 (9.8%)	11 (10.2%)			8 (9.1%)	
**T status**											0.043
T1		287 (61.7%)		89 (84.0%)		105 (64.4%)	55 (50.9%)			38 (43.2%)	
T2		157 (33.8%)		16 (15.1%)		53 (32.5%)	46 (42.6%)			42 (47.7%)	
T3-T4		21 (4.5%)		1 (0.9%)		5 (3.1%)	7 (6.5%)			8 (9.1%)	
**N status**											0.129
N0		262 (56.3%)		51 (48.1%)		124 (76.1%)	57 (52.8%)			30 (34.1%)	
N1		66 (14.2%)		4 (3.8%)		24 (14.7%)	21 (19.4%)			17 (19.3%)	
N2		34 (7.3%)		3 (2.8%)		15 (9.2%)	6 (5.6%)			10 (11.4%)	
**Pleural invasion**											<0.001
Yes		137 (29.5%)		15 (14.2%)		46 (28.2%)	43 (39.8%)			33 (37.5%)	
No		326 (70.1%)		90 (84.9%)		117 (71.8%)	64 (59.3%)			55 (62.5%)	
**Lymphovascular invasion**											<0.001
Yes		91 (19.6%)		7 (6.6%)		23 (14.1%)	34 (31.5%)			27 (30.7%)	
No		367 (78.9%)		94 (88.7%)		140 (85.9%)	73 (67.6%)			60 (68.2%)	
**Spread through air spaces (STAS) status**											<0.001
Absent		251 (54.0%)		70 (66.0%)		72 (44.2%)	48 (44.4%)			61 (69.3%)	
Present		214 (46.0%)		36 (34.0%)		91 (55.8%)	60 (55.6%)			27 (30.7%)	

### The genomic comparison of MPC versus non-MPC patients

**Figure 2:**
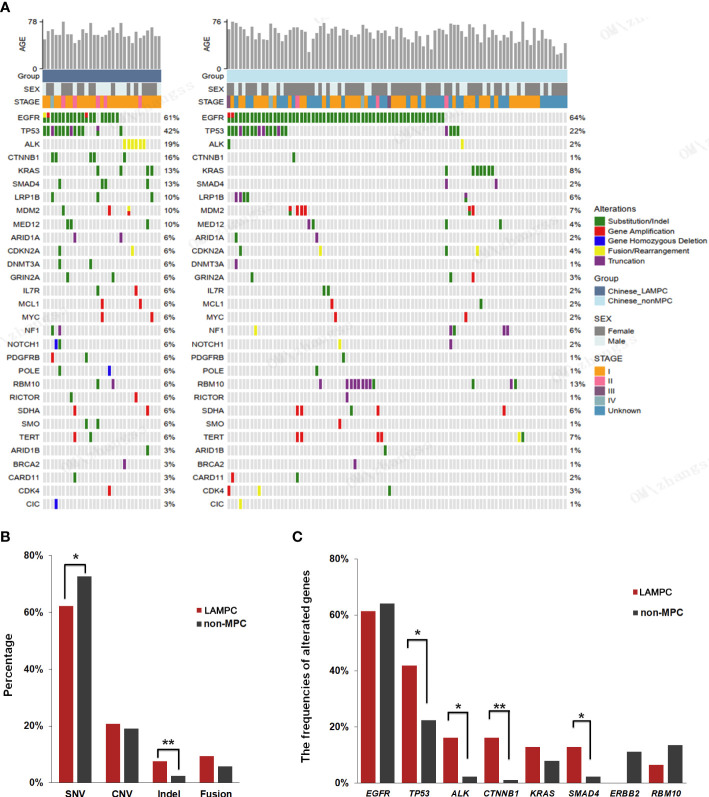
A comparison of genomic alterations between LAMPC and non-MPC patients. **(A)** A genomic alteration profile of 31 LAMPC samples from 29 patients and 89 non-MPC patients. **(B)** A comparison of mutation types between the two groups. **(C)** A comparison of major driver gene mutations between LAMPC and non-MPC groups. ^*^ *p* < 0.05, ^**^ *p* < 0.01.

To further explore the mechanism of malignant progression for tumors caused by MPC, molecular mechanisms related to tumor prognosis, and potential targets for effective treatment in patients with LAMPC, twenty-nine (20) LAMPC and 89 non-MPC patients that had undergone NGS testing were included in this analysis designed to compare differences in genomic characteristics between the LAMPC and non-MPC groups. Clinicopathological features for these 118 LA patients (including age, gender, tumor size, stage, T status, N status, pleural invasion, lymphovascular invasion and STAS) are summarized in **Table 2**. Higher proportion of LAMPC patients had pleural invasion, lymphovascular invasion and STAS than that patients with non-MPC. The 31 LAMPC samples from 29 MPC patients (two patients had two primary tumor samples) included 5 MPC-1, 13 MPC-2, 10 MPC-3, and 3 MPCs with an unknown proportion. NGS testing was performed for 29 MPC and 89 non-MPC patients. Two-hundred and ninety-six (296) genes were analyzed (**Figure 2A**). Gene mutations, including SNVs, indels, CNVs, and gene-gene fusions (FUS), were detected. Within the MPC cohort, a total of 159 somatic genes variants were detected. The most predominant variants were SNVs (62.3%, 99/158), CNVs (20.8%, 33/159), fusion/rearrangements (9.4%, 15/159), and indels (7.5%, 12/159). The frequency of SNVs was significantly lower (*p* = 0.01) in LAMPC patients as compared to non-MPC patients, while indels were more frequent within the LAMPC group than that in the non-MPC group (*p* < 0.01, **Figure 2B**). The top frequently altered genes in LAMPC patients were *EGFR* (61.3%), *TP53* (41.9%), *ALK* (19.4%), *CTNNB1* (16.1%), *SMAD4* (12.9%), and *KRAS* (12.9%). The most frequent alterations in non-MPC patients were *EGFR* (64%), *TP53* (22.5%), *RBM10* (13.5%), *ERBB2* (11.2%), *KRAS* (7.9%), *MDM2* (7%), and *TERT* (7%). *EGFR* mutations had comparable pooled mutation rates in the LAMPC and non-MPC groups (61.3% vs. 64.0%). We also determined that mutations in *TP53*, *ALK*, *CTNNB1*, and *SMAD4* were significantly enriched in LAMPC patients as compared to non-MPC patients (41.9% vs. 22.5%, *p* = 0.037; 16.1% vs. 2.2%, *p* = 0.012; 16.1% vs. 1.1%, *p* = 0.004; 12.9% vs. 2.2%, *p* = 0.038, respectively, **Figure 2C**). These genomic alterations may be potentially correlated with the formation of micropapillary structures. Mutations in *KRAS* also tended to have a higher frequency rate in LAMPC patients as compared to non-MPC patients (12.9% vs. 7.9%, *p* = 0.472, **Figure 2C**), although the rate was not statistically significant. *ERBB2* mutations were only detected in non-MPC patients, and *RBM10* mutations were detected more frequently in non-MPC than in LAMPC patients (13.5% vs. 6.5%, *p* = 0.516, **Figure 2C**).

**Table 2:** Clinicopathologic features of lung adenocarcinoma with and without micropapillary pattern.

	Total (n = 118)	MPC (n = 29)	Non-MPC (n = 89)
**Age**			
Median age, years (range)	556.5 (23-78)	56 (42-78)	57.5 (23-76)
**Gender**			
Male	41	14 (48.3%)	27 (30.3%)
Female	77	15 (51.7%)	62 (69.7%)
**Tumor size**			
Median size, cm (range)	1.5 (0.5-7)	2.1 (0.9-4.8)	1.4 (0.5-7)
**Stage**			
I	86	23 (79.3%)	63 (70.7%)
II	10	5 (17.2%)	5 (5.6%)
III-IV	9	1 (3.4%)	8 (9.0%)
UK	13	0 (0.0%)	13 (16.3%)
**T status**			
T1	85	24 (82.7%)	61 (68.5%)
T2	8	4 (13.8%)	4 (4.5%)
T3+T4	10	1 (3.4%)	9 (10.1%)
UK	15	0 (0.0%)	15 (16.9%)
**N status**			
N0	98	23 (79.3%)	75 (84.3%)
N1	7	6 (20.7%)	1 (1.1%)
N2	3	0 (0.0%)	3 (3.3%)
UK	10	0 (0.0%)	10 (11.2%)
**Pleural invasion**			
Yes	9	9 (31.0%)	0 (0.0%)
No	106	20 (69.0%)	86 (96.6%)
UK	3	0 (0.0%)	3 (3.4%)
**Lymphovascular invasion**			
Yes	2	2 (6.9%)	0 (0.0%)
No	113	27 (93.1%)	86 (96.6%)
UK	3	0 (0.0%)	3 (3.4%)
**Spread through air spaces (STAS) status**			
Absent	93 (78.8%)	13 (44.8%)	80 (89.9%)
Present	22 (18.6%)	16 (55.2%)	6 (6.7%)
UK	3 (2.5%)	0 (0.0%)	3 (3.4%)

In addition, the genomic alterations were also analyzed in MSKCC_MPC and MSKCC_non-MPC patients (**Table 3**). The top frequently altered genes in MSKCC_MPC patients were *TP53* (42.9%), *KRAS* (34.3%), *EGFR* (17.1%), *KEAP1* (17.1%), *NOTCH4* (17.1%) and *STK11* (17.1%). The most frequent alterations in MSKCC_non-MPC patients were *KRAS* (38.7%), *TP53* (36.2%), *EGFR* (31.4%), *RBM10* (17.4%), *STK11* (14.9%) and *KEAP1* (11.0%). High-frequency mutant genes were generally consistent between the MSKCC_MPC and MSKCC_non-MPC patients. We found that NOTCH4 mutation was significantly higher in the MSKCC_MPC group than in the MSKCC_non-MPC group (17.1% vs 3.6%, *p* = 0.003, **Table 4**).

**Table 3:** The top 26 genomic alterations in MSKCC_MPC and MSKCC_non-MPC patients.

MSKCC_MPC (n = 35)		MSKCC_non-MPC (n = 563)	
Gene	Frequency	Gene	Frequency
TP53	42.9%	KRAS	38.7%
KRAS	34.3%	TP53	36.2%
EGFR	17.1%	EGFR	31.4%
KEAP1	17.1%	RBM10	17.4%
NOTCH4	17.1%	STK11	14.9%
STK11	17.1%	KEAP1	11.0%
NTRK3	14.3%	FAT1	8.2%
ERBB4	11.4%	NF1	8.0%
IKZF1	11.4%	PTPRD	7.8%
PTPRT	11.4%	PTPRT	7.6%
SETD2	11.4%	ATM	7.3%
ATM	8.6%	SETD2	6.9%
BRAF	8.6%	BRAF	6.7%
CDC73	8.6%	MED12	6.7%
DNMT3B	8.6%	EPHA3	6.6%
HGF	8.6%	MET	6.4%
INHBA	8.6%	PIK3CA	6.2%
INPPL1	8.6%	MGA	5.7%
JAK3	8.6%	SMARCA4	5.5%
MTOR	8.6%	ATRX	5.3%
MYOD1	8.6%	EPHA5	5.3%
NF1	8.6%	NTRK3	5.2%
NOTCH2	8.6%	ARID2	5.0%
PTPRD	8.6%	CDKN2A	4.6%
RBM10	8.6%	ERBB2	4.6%
RPTOR	8.6%	TERT	4.6%

**Table 4:** A comparison of genomic alterations between MSKCC_MPC and MSKCC_non-MPC patients.

Gene	MSKCC_MPC_MUT	MSKCC_non-MPC_MUT	p-value
TP53	42.9%	36.2%	0.471
KRAS	34.3%	38.7%	0.721
NOTCH4	17.1%	3.6%	0.003
EGFR	17.1%	31.4%	0.089
KEAP1	17.1%	11.0%	0.270
STK11	17.1%	14.9%	0.633
RBM10	8.6%	17.4%	0.244
CTNNB1	5.7%	2.8%	0.284
SMAD4	2.9%	3.9%	1.000
ERBB2	2.9%	4.6%	1.000
ALK	0.0%	3.6%	0.623

### The signaling pathway analysis

**Figure 3:**
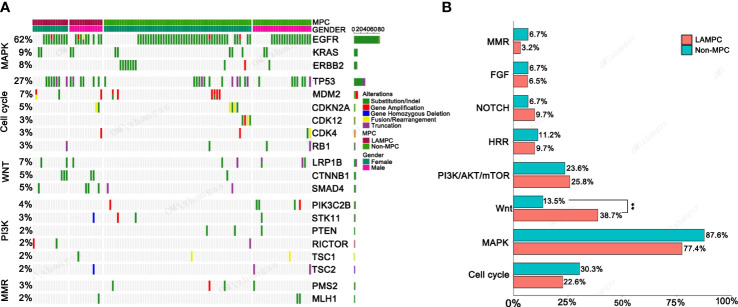
The genomic mutations involved in important signaling pathways in LAMPC and non-MPC patients. **(A)** The genomic alteration profile of pathways between LAMPC and non-MPC patients. **(B)** A comparison of the frequency of pathway changes between LAMPC and non-MPC patients. ^**^ *p* < 0.01.

Further enrichment using a signaling pathway analysis were performed in chinese_MPC patients and Memorial Sloan Kettering Cancer Center (MSKCC)_MPC patients. For chinese_MPC patients, 77.4% (24/31) harbored genomic alterations for the RTK/RAS/MAPK pathway, 22.6% (7/31) had cell-cycle pathway alterations, 38.7% (12/31) had Wnt pathway alterations, and 25.8% (8/31) had PI3K/AKT/mTOR pathway alterations (**Figures 3A, B**). The frequency of Wnt signaling pathway gene mutations was significantly higher in LAMPC patients as compared to non-MPC patients (38.7% vs. 13.5%, *p =* 0.002, **Figure 3B**). These results demonstrated that the Wnt alterations in signaling pathway may be involved in the regulatory mechanism of MPC formation.

### The correlation between genomic alterations and clinicopathological features in LAMPC

**Figure 4:**
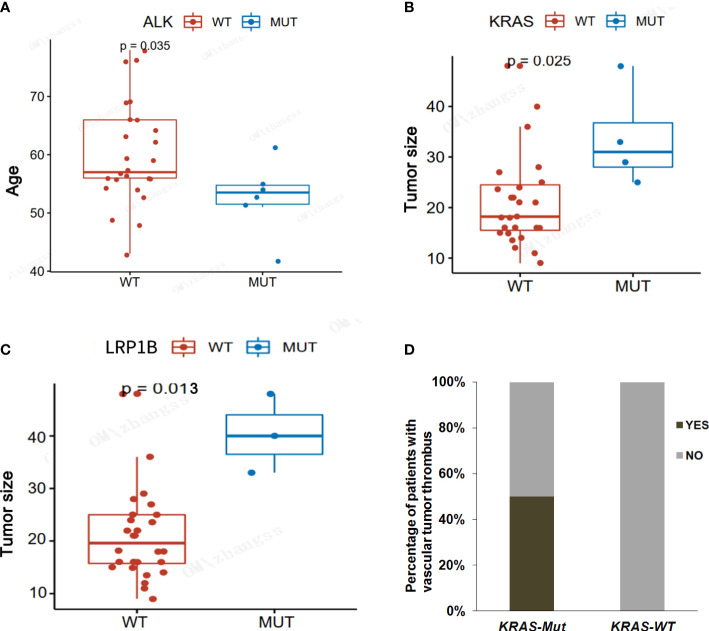
Correlation analyses between major driver gene alterations and clinicopathological characteristics in LAMPC patients. **(A)** A correlation analysis between *ALK* mutation and age. **(B)** A correlation analysis between *KRAS* mutation and tumor size. **(C)** A correlation analysis between *LRBP1* mutation and tumor size. **(D)** A correlation analysis between *KRAS* mutation and vascular tumor thrombus. *p*< 0.05 was considered statistically significant, and *p* < 0.01 was considered more statistically significant.

We also analyzed the correlation between major driver genes and clinicopathological features, including age, gender, tumor size, nerve membrane invasion, pleural invasion, and vascular tumor thrombus, in LAMPC patients. The results indicated that *ALK* fusion was significant in younger patients as compared to elderly patients (median: 52.7 vs. 60.1, *p =* 0.035, **Figure 4A** and **Table 5**). *KRAS* and *LBP1B* mutations were significantly associated with tumor size, and patients with *KRAS* or *LBP1B* mutations had significantly larger tumor diameters as compared to patients with wild-type (median: 3.1 vs. 1.8 cm, *p =* 0.025; median:4.0 vs 2.0 cm, *p* = 0.013, respectively **Figures 4B, C**, and **Table 5**). Vascular tumor thrombus was additionally found in two of four LAMPC patients with *KRAS* mutations, but not in patients with wild-type *KRAS*, suggesting that LAMPC patients with *KRAS* mutations are more likely to develop vascular tumor thrombus (*p =* 0.0129, **Figure 4D** and **Table 5**).

**Table 5:** Correlation analysis between major driver gene variants and clinical characteristics in patients with MPC.

	*EGFR*			*TP53*			*ALK*		
	WT	MUT	*p*	WT	MUT	*p*	WT	MUT	*p*
**Age**			0.290			0.659			0.035
n (median)	12 (57.33)	19 (59.47)		18 (58.22)	13 (59.23)		25 (60.08)	6 (52.67)	
**Gender**			1.000			1.000			1.000
Female	6	10		9	7		13	3	
male	6	9		9	6		12	3	
**Tumor size**			0.855			0.495			0.582
n (median)	12 (2.38)	19 (2.33)		18 (2.26)	13 (2.48)		25 (2.42)	6 (2.05)	
**Nerve invasion**			0.142			0.714			0.355
Yes	2	0		1	1		1	1	
No	10	19		17	12		24	5	
**Pleural invasion**			0.447			0.701			0.265
Yes	5	5		5	5		8	2	
No	7	14		13	8		17	4	
**Vascular tumor thrombus**			0.142			0.168			1.000
Yes	2	0		0	2		2	0	
No	10	19		18	11		23	6	
**Spread through air spaces (STAS) status**			1.000			0.294			0.676
Absent	5	8		6	7		10	3	
Present	7	11		12	6		15	3	
	***CTNNB1***			***KRAS***			***SMAD4***		
	WT	MUT	*p*	WT	MUT	*p*	WT	MUT	*p*
**Age**			0.628			0.081			0.723
n (median)	26 (58.12)	5 (61.40)		27 (57.56)	4 (66.00)		27 (58.89)	4 (57.00)	
**Gender**			1.000			1.000			0.333
Female	13	3		14	2		15	1	
male	13	2		13	2		12	3	
**Tumor size**			0.572			0.025			0.184
n (median)	25 (2.32)	6 (2.50)		27 (2.20)	4 (3.38)		27 (2.26)	4 (2.75)	
**Nerve invasion**			1.000			0.245			1.000
Yes	2	0		1	1		2	0	
No	24	5		26	3		25	4	
**Pleural invasion**			1.000			0.087			0.577
Yes	9	1		7	3		8	2	
No	17	4		20	1		19	2	
**Vascular tumor thrombus**			1.000			0.013			1.000
Yes	2	0		0	2		2	0	
No	24	5		27	2		25	4	
**Spread through air spaces (STAS) status**			1.000			0.621			0.284
Absent	11	2		12	1		10	3	
Present	15	3		15	3		17	1	

### The comparison of gene alterations in LAMPC between Chinese and Western populations

**Figure 5:**
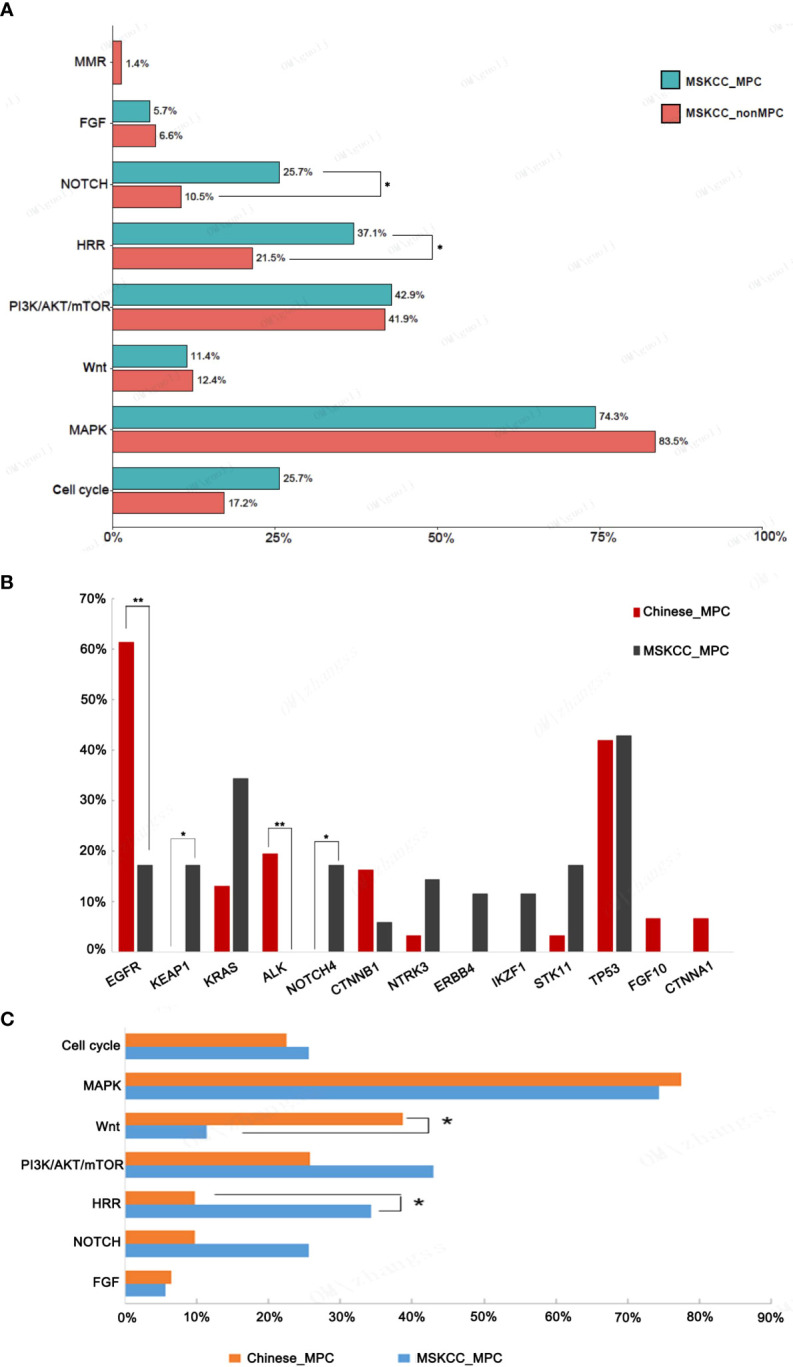
A comparison of gene mutations in the Chinese LAMPC and MSKCC LAMPC cohorts. **(A)** A comparison of the frequency of pathway changes between MSKCC_MPC and MSKCC_non-MPC patients. A comparison of frequencies for **(B)** main driver gene alterations and **(C)** pathways changes in the Chinese LAMPC and MSKCC LAMPC cohorts. ^*^ *p* < 0.05, ^**^ *p* < 0.01.

We analyzed targeted sequencing data generated from 35 MPC patients in a MSKCC cohort using the cBioPortal public database. Patients in this group included 12 males and 23 females. Seventy-seven percent (77.1%) of patients had an early stage tumor (Stage I-III). For MSKCC_MPC patients, 74.3% patients harbored genomic alterations for the MAPK pathway, and 42.9% patients harbored genomic alterations for the PI3K/AKT/mTOR pathway. Particularly, 37.1% and 25.7% MSKCC_MPC patients with alterations respectively observed in HRR and NOTCH pathway, which was significantly higher than that in MSKCC_nonMPC patients (37.1% vs. 21.5%, p = 0.037; 25.7% vs 10.5%, p = 0.012; **Figure 5A**). There are still differences in the pathways in which the genetic mutations are located between the Chinese_MPC and MSKCC_MPC cohorts. A partial gene analysis, based on the intersection of panel data, indicated that in the MSKCC cohort, the most frequently mutated genes were: *TP53* (42.9%), *KRAS* (34.3%), *NOTCH4* (17.1%), *STK11* (17.1%), *KEAP1* (17.1%), and *EGFR* (17.1%). A comparative analysis revealed that *EGFR* mutations were significantly higher in the Chinese cohort as compared to the MSKCC cohort (61.3% vs. 17.1%, *p* = 0.0003). *ALK* fusions were exclusively detected in the Chinese cohort, and mutations in *KEAP1* and *NOTCH4* were only detected in the MSKCC cohort (**Figure 5B**). A major signaling pathway analysis revealed that HRR-related pathway alterations were significantly higher in the MSKCC cohort as compared to the Chinese cohort (34.3% vs. 9.7%, *p* = 0.0207), while the frequency of Wnt pathway gene mutations was significantly higher in the Chinese cohort as compared to the MSKCC cohort (38.7% vs. 11.4%, *p* = 0.0196) (**Figure 5C**).

## Discussion

Micropapillary adenocarcinoma is one of the most aggressive histologic subtypes of lung adenocarcinoma. Patients with LAMPC have a high risk of early recurrence after surgery, and the risk for recurrence persisted over long term. Although the incidence of MPC-predominant LA is low, nearly half of LA patients have a minor proportion of MPC, which, for these patients, may contribute to a poor prognosis. Therefore, to better understand the biology of this subtype of LA and to develop effective treatments, a comprehensive analysis of genetic alterations for LAMPC is necessary.

Several previous studies have determined that the presence of at least a 5% MPC is inversely associated with survival (21, 22). Patients with sub-centimeter LA, with a ≥ 5% MPC, treated with a wedge resection, had a higher risk of recurrence (23). However, a MPC < 5% has also been determined to have a significant impact on OS (22). At present, the extent to which the percentage of MPC affects tumor progression is unclear. In our study, we determined that LA patients with a higher proportion of MPC had a higher tumor stage, pleural invasion, and vascular tumor thrombus formation. Our results are in-line with previous reports which indicate that a higher proportion of MPC is associated with a high degree of cancer aggressiveness, advanced stage, a high maximum standardized uptake value and distant metastasis, and a poor prognosis, even if MPC is not predominant (24, 25). In past studies, the presence of MPC, lymph node metastasis, pleural invasion, and gender have been shown to be associated with a poor prognosis (26).These researches suggested the increase in micropapillae component is a possible reflection of tumor progression secondary to accumulation of molecular alterations, and relate to the degree of tumor malignancy.

Surgical resection, chemotherapy, and targeted therapy are currently chief options for the treatment of LAMPC. At present, in patients with small size LAMPC, lobectomy is the best option for a potential cure (13). However, following the complete resection of Stage I LA, the presence of MPC is still associated with a poor prognosis (26).Comprehensive genomic profiles aid in our understanding of the MPC molecular mechanisms that lead to a poor prognosis, further helping us discover potential therapeutic targets aimed at providing more accurate individualized treatment for LAMPC. However, at present, only a few, incomplete studies containing a genomic comparison of LAMPC versus non-MPC exist. In such studies, *EGFR* mutations have been determined to be strongly associated with MPC-predominant subtypes, with prevalence rates of *EGFR*, *KRAS*, and *PIK3CA* mutations in LAMPC, respectively, at 76.0%, 6.0%, and 2.0% (27). In the same study, no *BRAF*, *NRAS*, *ALK*, *PDGFRA*, or other mutations were elucidated. *EGFR* mutations have also been determined to be more frequent in MPC-predominant LA patients than in non-MPC patients (28). We determined that the frequency of SNVs was significantly lower in LAMPC patients, whereas the frequency of indels was higher in LAMPC patients as compared to non-MPC patients. In our study, the most common mutated genes in LAMPC patients were *EGFR* (61.3%), *TP53* (41.9%), *ALK* (19.4%), *CTNNB1* (16.1%), *SMAD4* (12.9%), and *KRAS* (12.9%). *TP53*, *CTNNB1*, and *SMAD4* mutations, as well as *ALK* rearrangements/fusions, were significantly higher in LAMPC patients. *ERBB2* mutations were only detected in non-MPC patients. Our results are in contrast to a recent study by Zhang et al. (29), who determined similar mutation profiles and no significant differences in genomic alterations between LAMPC and non-MPC patients. We additionally found that *ALK* fusion was more frequent in younger patients, and that patients harboring mutations in *KRAS* or *LBP1B* had significantly larger tumor diameters as compared to patients with wild-type *KRAS*, suggesting that *KRAS* or *LBP1B* alterations are associated with LAMPC tumor progression. *TTF1*, *MTOR*, *BAI3*, and *CDKN2A* have been determined to be the most common mutated genes in Asian and European LAMPC patient cohorts (20). Mutations of *EGFR*, *KRAS*, and *BRAF* in LA, with at least a 75% MPC within a Western population, were investigated and 73% of patients were determined to harbor mutually exclusive mutations in one of these three genes (30). A Japanese study revealed that *EGFR* mutations are present in 40.1% of LAMPC (31). In our study, which employed the cBioPortal public database, *EGFR* mutations were determined to be significantly higher in the Chinese cohort as compared to the MSKCC cohort, *ALK* fusions were exclusively detected in the Chinese cohort, and *KEAP1* and *NOTCH4* mutations were only detected in the MSKCC_MPC cohort,and we suspect that NOTCH4 gene mutations may be a characteristic mutation unique to the MPC cohort of Western populations. Given the aggressive nature of LAMPC, our findings have implications for developing clinical therapeutic strategies to target LAMPC.

Our signaling pathway analysis revealed that in LAMPC the RTK/RAS/MAPK pathway, the cell-cycle pathway, the Wnt pathway, and the PI3K/AKT/mTOR pathway are altered. Wnt signaling pathway gene mutations were found to be significantly higher in LAMPC patients as compared to the non-MPC patients. The frequency of Wnt pathway gene mutations was also determined to be significantly higher in the Chinese cohort. Wnt signaling is key in regulating development and stemness, and has also been closely associated with cancer (32). Mutated Wnt pathway components can cause multiple growth-related pathologies and are frequent drivers in human cancers (33). For example, a past study revealed that WNT/β-catenin pathway activation *via WNT1* overexpression and *AXIN1* downregulation correlates with cadherin-catenin complex disruption and increased lymph node involvement in micropapillary-predominant LA (34). Such results suggest that the Wnt signaling pathway may be involved in regulatory mechanisms associated with MPC formation, and that it may play a role in the development of LAMPC.

In conclusion, we demonstrated that LA patients with a higher proportion of MPC tend to have a higher tumor stage, and are prone to pleural invasion and vascular tumor thrombosis formation, implying a potential link between MPC content and tumor malignancy. We comprehensively analyzed the characteristics of genomic alterations in LAMPC patients, and identified multiple new gene alterations and pathway changes associated with LAMPC. Our results provide more information regarding the nature of this cancer subtype and may aid future innovations related to drug-targeted gene alterations.

## Data availability statement

The raw data supporting the conclusions of this article will be made available by the authors, without undue reservation.

## Ethics statement

This study was approved by the Ethics Committee of the Affiliated Hospital of Qingdao University (Ethics No.: QYFYKYLL: 999811920), and all patients signed an informed consent form. The patients/participants provided their written informed consent to participate in this study.

## Author contributions

Project development: YW, PL, LL, and DW. Data collection: RY, YX, YH and JW. Data analysis: PL, LZ and SZ. Manuscript writing/editing: YW, PL, LL, DW, RY, YX, YH, JW and LG. All authors contributed to the article and approved the submitted version.

## Funding

This study was supported by funding from Wu Jieping Medical Foundation (320.6750.2021-01-4).

## Conflict of interest

LG, SZ and LZ are employees of OrigiMed. 

The remaining authors declare that the research was conducted in the absence of any commercial or financial relationships.

## Publisher’s note

All claims expressed in this article are solely those of the authors and do not necessarily represent those of their affiliated organizations, or those of the publisher, the editors and the reviewers. Any product that may be evaluated in this article, or claim that may be made by its manufacturer, is not guaranteed or endorsed by the publisher.
